# Single‐Cell Characterization of Terminal States and State‐Specific Transcriptional Regulatory Networks in Hepatocellular Carcinoma

**DOI:** 10.1111/jcmm.71214

**Published:** 2026-05-25

**Authors:** Biaolong Deng, Ke Xu, Yun Zhu, Wenhao Lin, Lang Lu, Tao Yang, Xiaoyan Zhou, Yuanyuan Chen

**Affiliations:** ^1^ The First Affiliated Hospital of Guangzhou Medical University Guangzhou China; ^2^ College of Science Nanjing Agricultural University Nanjing China

**Keywords:** cell fate, differentiation, hepatocellular carcinoma, transcriptional regulatory networks

## Abstract

Hepatocellular carcinoma (HCC) exhibits pronounced cellular heterogeneity and dynamic state transitions during tumour progression, yet the emergence of tumour cell states along fate trajectories and their transcriptional regulation remain unclear. Here, we established an integrated framework linking cell fate dynamics with transcriptional regulatory networks using publicly available single‐cell RNA sequencing data. Malignant cells were identified by inferred copy number variation, followed by CellRank‐based fate inference and SCENIC‐based regulatory network reconstruction. We identified three terminal HCC cell states—immune‐activated, metabolic, and proliferative hepatocytes—forming a differentiation continuum from stem‐like to more mature phenotypes. Each state was governed by a distinct regulatory network with specific core regulators, including IRF3, PPARA, and JUN. Integration with TCGA‐LIHC bulk transcriptomic and clinical data revealed that a proliferative state–derived transcriptional signature was associated with poorer overall survival. Together, our study provides a mechanistic framework linking tumour cell fate trajectories, regulatory heterogeneity and clinical outcomes in HCC.

## 1 Introduction

Cancer remains a major global public health challenge, with liver cancer representing one of the leading causes of cancer‐related mortality worldwide. In China, liver cancer ranked third in incidence in 2022, while its annual mortality rate was second only to lung cancer. A global liver cancer report published on 28 July 2025, in The Lancet by Jian Zhou, Jia Fan and colleagues from Zhongshan Hospital of Fudan University highlighted the continuously increasing global burden of liver cancer, identifying China as the country bearing the heaviest disease burden and accounting for 42.4% of all liver cancer cases worldwide. Without effective intervention strategies, the global burden of liver cancer is projected to double by 2050 [1]. Therefore, elucidating the mechanisms underlying liver cancer initiation and progression has become a central focus of contemporary cancer research, and improvements in prevention and treatment strategies are closely linked to the effectiveness of national public health systems. In China, the persistently high incidence of liver cancer, together with its insidious onset, low early diagnostic rate, and limited therapeutic options, results in poor overall prognosis and imposes a substantial burden on both society and families. Accordingly, identifying screening and therapeutic strategies that are compatible with national healthcare resources and population health literacy is of great practical significance for improving liver cancer prevention and control.

The development of single‐cell RNA sequencing (scRNA‐seq) technologies has provided unprecedented resolution for systematically characterizing cellular heterogeneity in hepatocellular carcinoma (HCC) [2]. Previous studies have leveraged scRNA‐seq to delineate the cellular composition of HCC tissues and to characterize transcriptional differences between malignant and non‐malignant cells [3]. However, most existing studies remain largely confined to static descriptions of cell types or subpopulations. Systematic analyses of tumour cell state transitions within continuous transcriptional space, the identification of terminal cell fates, and the potential fate relationships among distinct cell states remain limited. In particular, the transcriptional regulatory network architectures underlying different tumour cell states, as well as their key regulatory factors, have yet to be comprehensively elucidated.

In studies of the molecular mechanisms of liver cancer, gene regulatory network analysis has emerged as a powerful approach for systematically uncovering gene–gene interactions and identifying core regulatory factors that play critical roles in tumour initiation and progression. Depending on regulatory hierarchy and mode of action, gene regulatory networks can be classified into several categories, including gene co‐expression networks, miRNA–gene regulatory networks, transcription factor–gene (TF–gene) regulatory networks and transcription factor–miRNA (TF–miRNA) regulatory networks, which together constitute a complex and finely tuned transcriptional regulatory system [4, 5, 6, 7]. Among these, transcription factors directly control the expression of downstream target genes, thereby determining cellular transcriptional states and functional phenotypes. Consequently, TF–gene regulatory networks have become a major focus in molecular oncology research.

Nevertheless, regulatory networks inferred from bulk transcriptomic data often obscure pronounced intratumoral heterogeneity and are insufficient for resolving dynamic regulatory mechanisms across distinct cellular states or differentiation stages. Integrating cell fate inference with transcriptional regulatory network reconstruction enables the investigation of tumour cell state transitions at single‐cell resolution and provides deeper insights into the evolutionary processes of HCC. Accumulating evidence suggests that HCC initiation and progression are closely associated with tumour cell differentiation status and stemness properties. Tumour cells exhibiting high stemness features typically display enhanced proliferative capacity, therapeutic resistance, and invasive potential and are widely considered key drivers of HCC progression and recurrence [8]. However, at the single‐cell level, the continuous variation in differentiation and stemness among distinct tumour cell states, as well as the corresponding transcriptional regulatory mechanisms, remain poorly characterized.

Based on this background, the present study analyzes single‐cell transcriptomic data to systematically investigate the heterogeneity of HCC cells. Malignant and non‐malignant cells were first distinguished by copy number variation inference, followed by batch‐corrected integration of HCC cells. CellRank was then applied to model tumour cell fate trajectories and to identify three terminal cell fates with distinct biological characteristics [9, 10]. Subsequently, fate‐specific transcription factor–gene regulatory networks were reconstructed using Single‐Cell Regulatory Network Inference and Clustering (SCENIC) [11], enabling the systematic identification of core regulatory factors and their potential functions across different terminal fates. By further integrating stemness‐related gene program analyses and functional enrichment results, this study delineates the transcriptional regulatory basis of HCC heterogeneity from multiple perspectives, including cell state, fate trajectory and regulatory mechanism, thereby providing new insights into the initiation and progression of HCC.

## 2 Materials and Methods

**FIGURE 1:**
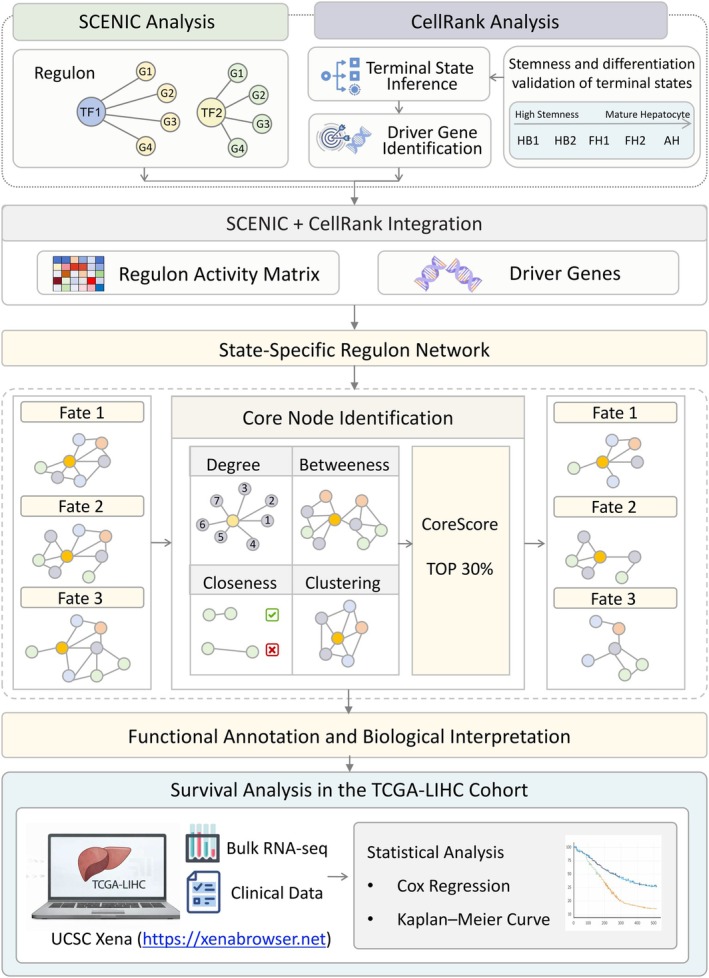
Overview of the analytical workflow of this study. Single‐cell transcriptional regulatory units (regulons) were inferred using SCENIC, while terminal cell states and fate‐driving genes were identified using CellRank. Regulon activity matrices were integrated with fate‐driving genes to construct state‐specific regulatory networks. Network topological features, including degree, betweenness, closeness and clustering coefficient, were combined into a composite CoreScore to identify core regulatory nodes (top 30%) for each terminal state. The resulting state‐specific networks were subjected to functional annotation and biological interpretation. Bulk RNA‐seq and clinical data from the TCGA‐LIHC cohort were further incorporated to evaluate the clinical relevance of single‐cell–derived transcriptional programs using survival analyses.

An overview of the single‐cell analysis workflow is shown in Figure 1.

### 2.1 Data Sources and Preprocessing

This study integrated single‐cell RNA sequencing data and bulk transcriptomic data to investigate cellular heterogeneity in hepatocellular carcinoma (HCC) and to evaluate the clinical relevance of single‐cell–derived transcriptional programs at the patient level. Single‐cell RNA sequencing data were used to characterize malignant cell states and transcriptional regulatory networks, while bulk RNA sequencing data from an independent cohort were used for clinicopathological and survival association analyses.

Single‐cell analyses were performed using a publicly available dataset (GSE149614), which comprises single‐cell RNA sequencing data from 10 HCC patients, including samples from primary tumours, portal vein tumour thrombi, metastatic lymph nodes and adjacent non‐tumour liver tissues [12]. Raw gene expression matrices were subjected to normalization and quality control following standard single‐cell RNA‐seq preprocessing procedures.

To distinguish malignant HCC cells from normal hepatocytes, copy number variation (CNV) profiles were inferred at single‐cell resolution using inferCNV. Well‐annotated normal hepatocytes were selected as the reference population to identify large‐scale chromosomal amplifications or deletions by comparing relative expression intensities across genomic regions. Cells exhibiting pronounced CNV deviations were classified as malignant tumour cells, whereas cells with stable CNV patterns similar to the reference were classified as non‐malignant cells and retained for downstream analyses.

To integrate multi‐sample single‐cell RNA‐seq data and reduce systematic bias introduced by sample origin, malignant and non‐malignant cells were first identified. The expression matrix was then normalized, highly variable genes were selected and principal component analysis (PCA) was performed. Batch correction was subsequently carried out using Harmony to align cells from different samples in a shared low‐dimensional space while preserving biological variation. The Harmony‐corrected embeddings were used for downstream analyses, including UMAP visualization, cell fate inference and transcriptional regulatory network reconstruction.

To quantitatively assess the quality of integration, we calculated two per‐cell neighbourhood‐based metrics in the UMAP space: a batch mixing score and a tissue type separation score. Both metrics were computed based on the 30 nearest neighbours of each cell. The batch mixing score was defined as the proportion of nearest neighbours originating from different samples, reflecting the degree of inter‐sample mixing in the integrated space. Higher values indicate better mixing and reduced batch effects. The tissue type separation score was defined as the proportion of nearest neighbours not belonging to the same tissue type, reflecting the extent of separation between cell populations. Lower values indicate tighter clustering within the same tissue type and better preservation of tissue‐specific structure. All scores were stored in the metadata for subsequent visualization and comparative analysis across tissue types.

Bulk RNA sequencing data together with corresponding clinical and overall survival information were obtained from The Cancer Genome Atlas Liver Hepatocellular Carcinoma (TCGA‐LIHC) cohort via the UCSC Xena platform (https://xenabrowser.net). These data were used for patient‐level analyses, including the construction of transcriptional signatures derived from single‐cell analyses and assessment of their associations with clinicopathological characteristics and overall survival. Detailed analytical procedures for TCGA‐based analyses are described in a separate section.

### 2.2 Terminal State Inference and Identification of Fate‐Driving Genes

Cell fate trajectories and fate‐driving genes were inferred using CellRank [9, 10], which enables dynamic fate inference based on transcriptional similarity and continuous state transitions in single‐cell transcriptomic space. CellRank integrates low‐dimensional embeddings to construct a directed transition probability matrix between individual cells, formalizing cell fate evolution as a Markov chain process, in which each cell represents a distinct state node.

To characterize the relative progression of cells along continuous fate trajectories, pseudotime information was incorporated to provide an objective temporal ordering of state transitions. Based on the normalized expression matrix, CellRank modelling identified the terminal HCC cell states exhibiting stable dynamical properties.

To identify molecular regulators associated with terminal state formation and maintenance, CellRank‐inferred probability flow analysis and null distribution testing were applied. Fate‐driving genes significantly enriched toward each terminal state were identified using a false discovery rate (FDR) threshold of < 0.05. These state‐specific fate‐driving gene sets served as the molecular basis for subsequent construction of state‐specific transcriptional regulatory networks.

### 2.3 Assessment of Hepatocyte Differentiation Status and Stemness

To quantitatively assess heterogeneity in differentiation status and stemness among the three terminal states, experimentally validated hepatocyte differentiation‐related gene programs from previous studies were incorporated [8]. These programs cover sequential stages along hepatocyte development: HB1 (hepatoblast stage 1, early liver progenitor cells), HB2 (hepatoblast stage 2, late liver progenitor cells), FH1 (fetal hepatocyte stage 1), FH2 (fetal hepatocyte stage 2), and AH (adult hepatocyte, mature liver cells), representing the continuous progression from early progenitors to fully differentiated, mature hepatocytes.

Based on the normalized single‐cell expression matrix, the AUCell algorithm was used to calculate activity scores for each differentiation program at single‐cell resolution. To reduce potential noise from ambiguous fate assignments, subsequent analyses were restricted to cells confidently assigned by CellRank to one of the three terminal states. AUCell score distributions were visualized in UMAP embedding space, and two‐sided Wilcoxon rank‐sum tests were applied to evaluate statistical differences in program activity among terminal states.

This analytical framework was adapted from the hepatocyte differentiation assessment system established by Wesley et al. in a single‐cell atlas of human liver development, in which the core gene sets were experimentally validated through immunofluorescence staining, functional enrichment analysis, and in vitro differentiation assays [8].

### 2.4 Single‐Cell Transcriptional Regulatory Network Reconstruction

To investigate transcriptional regulatory mechanisms underlying HCC cell state heterogeneity, we applied the SCENIC framework implemented in R (R SCENIC) to reconstruct single‐cell transcriptional regulatory networks. The analysis was performed in three main steps: (i) construction of gene regulatory networks using the GENIE3 algorithm (v1.26.0); (ii) motif enrichment analysis through RcisTarget to validate transcription factor (TF)–target gene interactions; and (iii) quantification of regulon activity at the single‐cell level using AUCell (v1.26.0). The list of transcription factors was obtained from the built‐in RcisTarget databases provided in the SCENIC workflow. Regulons, defined as functional regulatory units consisting of a TF and its directly regulated target genes, were thereby established. The resulting regulon activity matrix served as the foundation for subsequent analyses of regulatory heterogeneity.

To evaluate the influence of each regulon within the network, the Regulon Specificity Score (RSS) was calculated for each regulon across cell states [13]. RSS quantifies the specificity of a regulon to a particular cell state, with higher values indicating stronger state‐specific activity. Mathematically, RSS is defined as:

$$\mathrm{RSS}=1-\mathrm{JSD}\left(P^{R},P^{C}\right)$$

where $(P^{R}=\left(P_{1}^{R},\ldots ,P_{n}^{R}\right)$ denotes the probability distribution of regulon activity across all cells, satisfying $\sum_{i=1}^{n}P_{i}^{R}=1$ and $(P^{C}=\left(P_{1}^{C},\ldots ,P_{n}^{C}\right)$ represents the cell‐type indicator distribution, where each is an indicator function:

$$P_{i}^{C}=\left\{\begin{array}{c} 0\;,\text{if}\;{\text{cell}}_{i}\notin {\text{celltype}}^{C}\\ 1,\text{if}\;{\text{cell}}_{i}\in {\text{celltype}}^{C} \end{array}\right.$$

and $\sum_{i=1}^{n}P_{i}^{C}=1$. Here, $\mathrm{JSD}\left(P^{R},P^{C}\right)$ is the Jensen‐Shannon divergence between the two distributions, computed as:

$$\mathrm{JSD}\left(P^{R},P^{C}\right)=h\left(\frac{P^{R}+P^{C}}{2}\right)-\frac{H\left(P^{R}\right)+H\left(P^{C}\right)}{2}$$

where $H\left(P\right)=-\sum P_{i}\log\left(P_{i}\right)$ is the Shannon entropy. This formulation ensures that RSS reflects how concentrated the regulon's activity is within a specific cell type relative to its overall distribution.

To further characterize the global regulatory architecture, the Connection Specificity Index (CSI) was calculated to quantify cooperative relationships among regulons. CSI measures how specific the correlation between a pair of regulons is relative to all other regulon pairs. For two regulons and, we compute the Pearson correlation of their activity profiles across cells. We then compare this value to the correlations of and with every other regulon $i\left(i\neq A,B\right)$. An indicator $m_{i}^{A,B}$ is set to 1 if either $\mathrm{PCC}\left(A,i\right)<\mathrm{PCC}\left(A,B\right)$ or $\mathrm{PCC}\left(B,i\right)<\mathrm{PCC}\left(A,B\right)$ holds, and 0 otherwise. CSI is defined as the proportion of such cases:

$$\mathrm{CSI}\left(A,B\right)=\frac{\sum_{I\neq A,B}m_{i}^{A,B}}{{\left(N-2\right)}^{2}}$$

where N is the total number of regulons. CSI values range from 0 to 1, with higher values indicating that the co‐activity pattern between A and B is highly specific and suggestive of potential functional cooperation.

### 2.5 Identification of State‐Specific High‐Activity Regulons

To characterize transcriptional regulatory remodelling during differentiation toward distinct terminal states, cells were grouped according to the three CellRank‐defined terminal states. For each regulon, mean AUC scores were calculated within each state to represent average activity levels. Row‐wise *Z*‐score normalization was applied to minimize intrinsic activity differences across regulons.

Regulons exhibiting significantly elevated activity in one terminal state compared with others were identified using strict criteria (|*Z*‐score| > 1.5 and *p* < 0.05, Wilcoxon rank‐sum test). These regulons were defined as state‐specific high‐activity regulons and served as key regulatory units for downstream network construction and mechanistic analysis.

### 2.6 Core Regulatory Node Identification and Network Analysis

To focus on transcriptionally active regulators involved in fate determination, state‐specific high‐activity regulon target genes were intersected with corresponding CellRank‐derived fate‐driving gene sets. Genes meeting both criteria were defined as core genes.

Based on this intersection, compact state‐specific regulatory networks were constructed. Network topological properties were calculated using the NetworkX toolkit, including in‐degree, out‐degree, betweenness centrality, closeness centrality and clustering coefficient. To comprehensively evaluate node importance, these metrics were standardized using *Z*‐score normalization and equally weighted to generate a composite CoreScore. The equal weighting was chosen to avoid subjective bias and to ensure that each topological aspect contributes uniformly to the assessment of regulatory importance. Nodes ranking within the top 30% of CoreScore values were defined as core regulatory nodes.

### 2.7 Functional Enrichment Analysis

To elucidate biological functions associated with core regulatory mechanisms in each terminal state, Gene Ontology (GO) and Kyoto Encyclopedia of Genes and Genomes (KEGG) pathway enrichment analyses were performed using the R package clusterProfiler. GO annotations included biological process (BP), molecular function (MF) and cellular component (CC) categories, while KEGG analysis focused on key signalling and metabolic pathways.

Multiple testing correction was performed using the Benjamini–Hochberg method, with an adjusted *p* value < 0.05 defined as the threshold for statistical significance. Comparative analysis of enriched terms across terminal states enabled systematic delineation of state‐specific biological processes and signalling pathways.

### 2.8 Construction of the Fate2 Regulatory Signature and Clinical Association Analysis

To evaluate the clinical relevance of the Fate2 (malignant proliferative) hepatocyte state at the patient level, we first curated a set of core transcription factors from the Fate2 regulatory network identified by single‐cell transcriptomic analysis. The expression prevalence and expression intensity of these transcription factors were compared across Fate0, Fate1, and Fate2 hepatocyte states to characterize the activation patterns of the Fate2 regulatory program in distinct cellular contexts.

Subsequently, a Fate2 signature was constructed in the TCGA‐LIHC cohort. Specifically, expression values of the 26 transcription factors comprising the Fate2 regulatory network were standardized across samples using gene‐wise *Z*‐score normalization, and the mean *Z*‐score of these genes was calculated for each patient to derive the Fate2 signature score. The signature score was then integrated with AJCC pathological stage and overall survival (OS) data. Associations between the Fate2 signature and tumour stage were assessed using non‐parametric tests, while prognostic relevance was evaluated using Cox proportional hazards regression and Kaplan–Meier survival analysis. Continuous‐variable Cox regression was used as the primary analytical approach, with Kaplan–Meier analysis applied for visualization purposes.

### 2.9 Artificial Intelligence Declaration

In the preparation of this manuscript, we used [ChatGPT 5.0] to assist with language translation and manuscript polishing. The AI tool was used solely to improve the clarity and readability of the text. All content generated by the AI was reviewed, revised, and approved by the authors, who take full responsibility for the final content of the manuscript.

## 3 Results

### 3.1 HCC Cells Exhibit Three Terminal Cell States With Distinct Differentiation Trajectories at Single‐Cell Resolution

CellRank analysis identified stable terminal states characterized by high attractor properties during long‐term cell state transitions, representing fate endpoints toward which HCC cells converge during state evolution. Based on steady‐state probability estimation, three terminal cell states were consistently identified within the HCC cell population and were designated as fate 0, fate 1, and fate 2.

**FIGURE 2:**
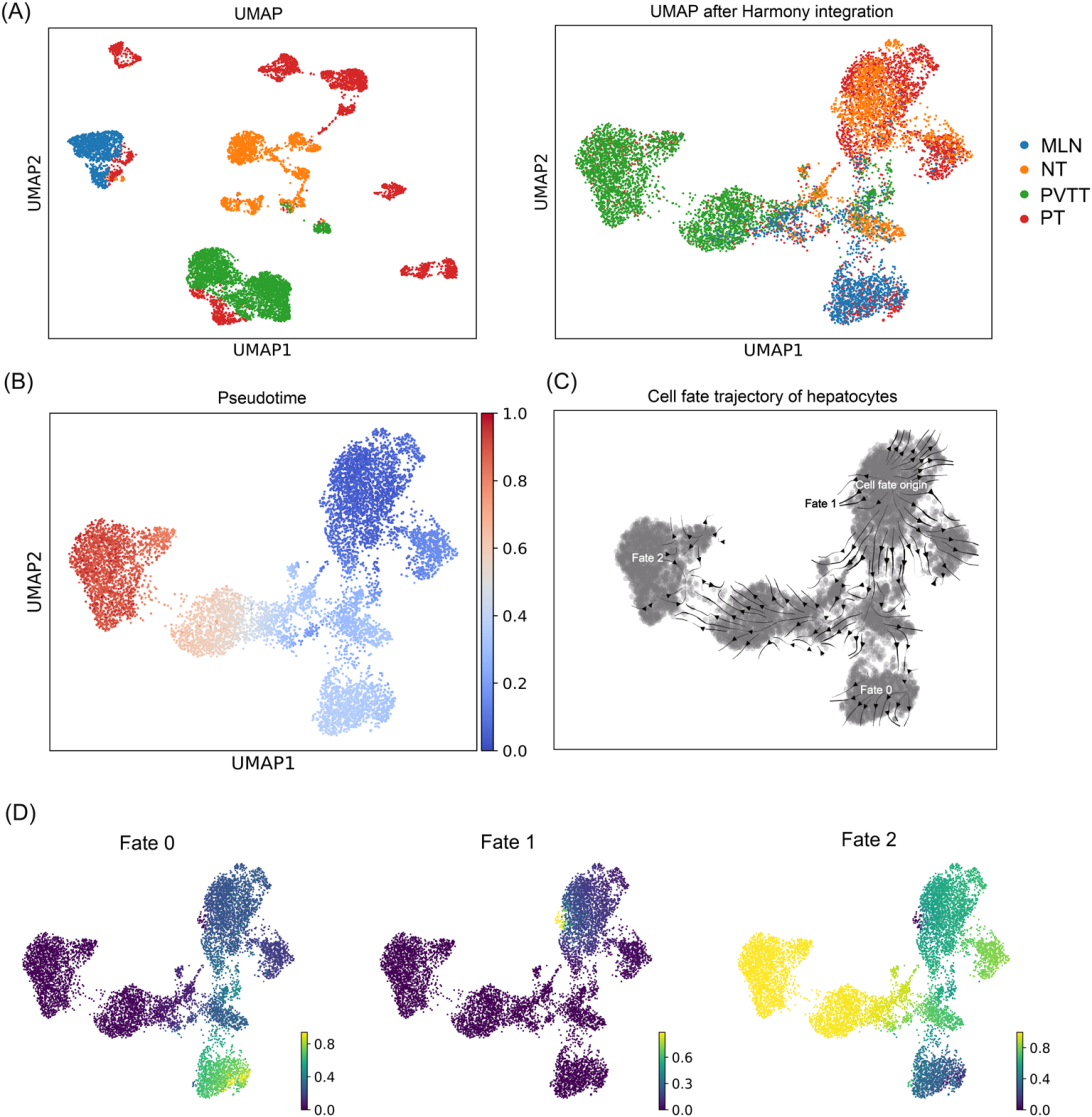
Single‐cell transcriptomic clustering, batch correction, and fate state inference of hepatocytes and HCC cells. (A) UMAP visualization of single‐cell transcriptomes before batch correction (left) and after Harmony‐based integration (right). Cells are coloured according to tissue origin, including metastatic lymph nodes (MLN), portal vein tumour thrombi (PVTT), primary tumour tissues (PT), and non‐tumour liver tissues (NT). (B) Distribution of CellRank‐inferred pseudotime values across the UMAP embedding, with the colour gradient from blue to red indicating progression from early to late pseudotime states. (C) Cell fate trajectories of hepatocytes and HCC cells inferred by CellRank. Arrow directions indicate potential state transition trends toward distinct terminal states. (D) UMAP‐based probability distributions of the three terminal fates (Fate 0,1,2), with the colour gradient from blue to yellow/green indicating increasing assignment probability to the corresponding terminal state.

Low‐dimensional visualization showed that, prior to batch correction, cells derived from different tissue sources—including metastatic lymph nodes (MLN), portal vein tumour thrombi (PVTT) and primary tumours (PT)—exhibited dispersed distributions in UMAP space (Figure 2A, **left**). After Harmony‐based integration, cells with similar biological characteristics displayed tighter aggregation (Figure 2A, **right**), indicating effective removal of batch effects and providing a unified expression landscape for downstream analyses. Using the batch‐corrected data, CellRank‐inferred pseudotime values formed a continuous gradient across the UMAP embedding (Figure 2B), with lower pseudotime values corresponding to early‐stage cells and higher values indicating cells approaching terminal states.

Global fate trajectories inferred by CellRank revealed a convergent, multi‐branch architecture with directional transitions toward multiple endpoints (Figure 2C), suggesting that HCC cells undergo diversification from an initial state toward distinct terminal states through alternative differentiation pathways. Consistently, the spatial distributions of terminal state probabilities within UMAP space (Figure 2D) demonstrated pronounced region‐specific enrichment for each terminal state. These results indicate that the three terminal states are not randomly distributed but instead correspond to functionally differentiated subpopulations within the HCC cell population.

To evaluate the effectiveness of data integration, we next examined per‐cell batch mixing and tissue type separation scores in the Harmony‐corrected UMAP space (Figure S1A–B). Overall, batch mixing scores were consistently high across all tissue types, including tumour, lymph node, normal tissue, and portal vein tumour thrombus (PVTT), indicating that cells from different samples were well intermingled after integration and that batch effects were effectively minimized. In contrast, tissue type separation scores showed a more heterogeneous pattern across different tissues. Lymph node, normal tissue, and PVTT‐derived cells generally exhibited relatively high separation scores, suggesting more dispersed neighbourhood structures in the integrated space. Notably, tumour cells displayed significantly lower separation scores compared with other tissue types, indicating that tumour cells formed more compact neighbourhoods in UMAP space. This tighter clustering of tumour cells likely reflects stronger transcriptional coherence within the malignant population, which is consistent with their more homogeneous malignant state compared to non‐tumour tissues. Taken together, these results suggest that while Harmony effectively removed sample‐associated batch effects across all tissues, tumour cells retain a distinct and more cohesive transcriptional structure. These observations are consistent with the UMAP visualization before and after integration (Figure 2A) and support the reliability of downstream analyses, including cell fate inference and transcriptional regulatory network reconstruction.

The three terminal states are clearly non‐random and correspond to functionally distinct subpopulations within the HCC cell population. Fate 1 lies closest to normal‐like cells, suggesting a less aggressive phenotype, while Fate 2 is furthest from normal‐like cells and exhibits the strongest malignant features. Fate 0 falls between these two states. Overall, this arrangement reflects a directional gradient of malignancy, indicating that HCC cells follow heterogeneous differentiation paths while maintaining distinct terminal identities. This ordering further suggests a potential transitional trajectory among the three terminal states. Specifically, Fate 1 may represent a relatively early or less malignant state, which progresses through an intermediate state represented by Fate 0, and ultimately reaches the more aggressive Fate 2. This continuum from Fate 1 to Fate 0 to Fate 2 is consistent with a gradual increase in malignant features, supporting the notion of a stepwise progression rather than abrupt transitions between discrete states.

### 3.2 Terminal Cell States Exhibit a Continuous Gradient Along Hepatocyte Differentiation and Stemness Programs

To characterize the functional maturity associated with the terminal cell states inferred by CellRank, AUCell was used to calculate single‐cell activity scores for hepatocyte differentiation‐related gene programs (HB1, HB2, FH1, FH2 and AH) (Table S2). These programs represent sequential stages along the hepatocyte differentiation axis, ranging from hepatoblast‐like states through foetal hepatocyte stages to adult hepatocyte phenotypes.

**FIGURE 3:**
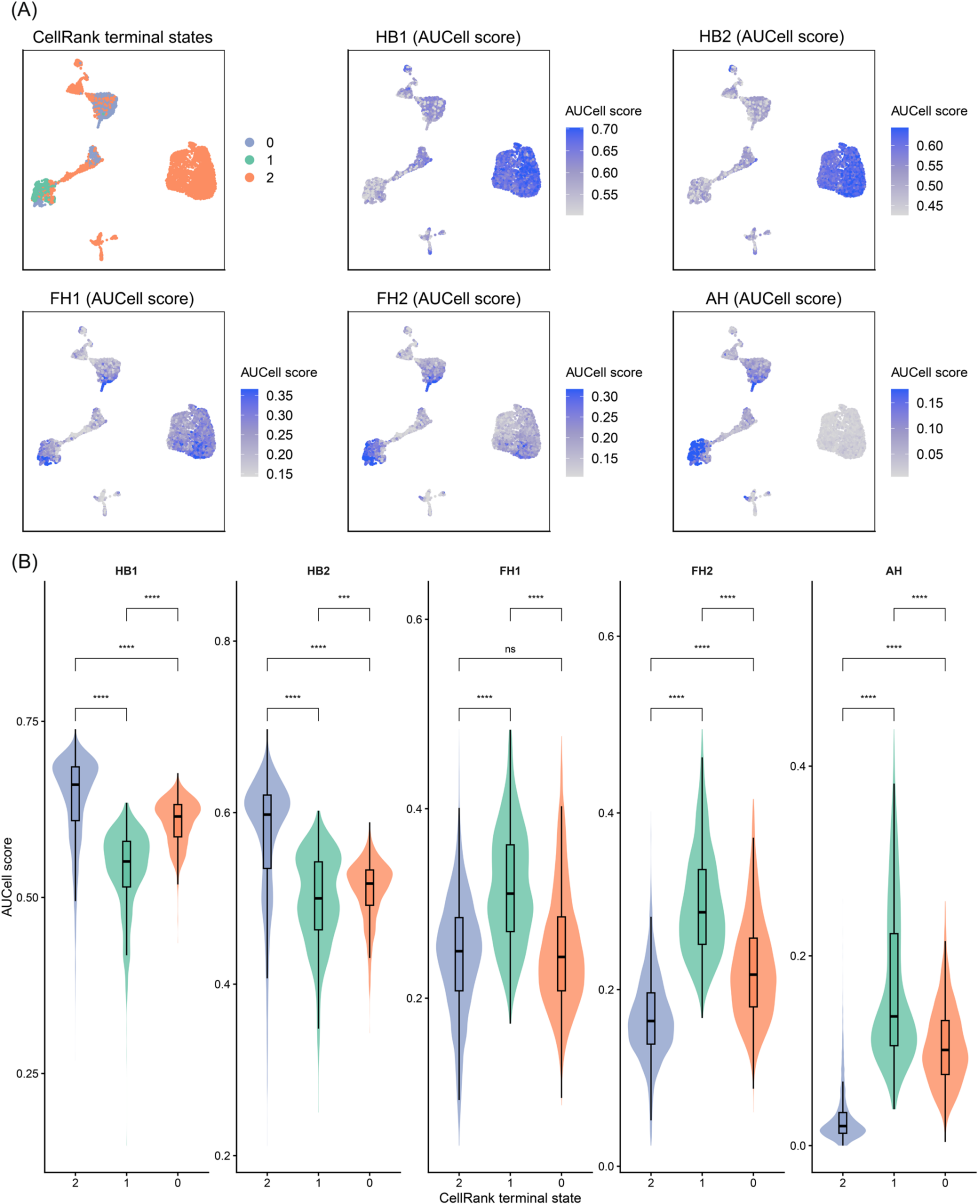
Distribution of hepatocyte differentiation program activities across three terminal cell states. (A) UMAP visualization of the three terminal cell states identified by CellRank. Colours indicate State 0 (stem‐like), State 1 (metabolic) and State 2 (proliferative). AUCell activity scores of hepatocyte differentiation‐related gene programs (HB1, HB2, FH1, FH2 and AH) are overlaid on the same low‐dimensional space. Colour gradients from light to dark correspond to increasing program activity, reflecting higher expression of the corresponding gene modules in each cell. (B) Violin plots showing AUCell activity scores of HB1, HB2, FH1, FH2 and AH programs across the three terminal cell states. Gene programs HB1 and HB2 correspond to hepatocyte early differentiation modules, FH1 and FH2 correspond to hepatocyte functional modules, and AH corresponds to adult hepatocyte maturation program (see Methods for details).

Visualization of program activity distributions in low‐dimensional space revealed pronounced stratification of differentiation programs across the three CellRank‐defined terminal states (Figure 3A). Cells assigned to state 2 exhibited markedly elevated AUCell scores for the HB1 and HB2 programs, which are associated with early hepatoblast‐like features, indicating the retention of immature, high‐stemness characteristics. In contrast, cells in state 1 showed enrichment of FH1 and FH2 program activity, corresponding to fetal hepatocyte features and suggesting a transitional maturation stage characterized by progressive acquisition of hepatocyte functions. Conversely, cells in state 0 displayed significantly higher activity of the AH program, indicative of a phenotype closely resembling functionally mature adult hepatocytes.

Statistical comparison of differentiation program activity at the terminal‐state level further supported these observations (Figure 3B). Along the HB–FH–AH differentiation axis, the three terminal states exhibited a continuous and largely non‐overlapping distribution pattern. Specifically, median HB1 and HB2 scores were significantly higher in state 2 than in states 0 and 1 (*p* < 0.001, Wilcoxon rank‐sum test, two‐sided). FH1 and FH2 scores were significantly elevated in state 1 compared with states 2 and 0 (*p* < 0.001), although FH1 did not differ significantly between states 0 and 2. In contrast, AH scores were significantly higher in state 0 than in states 1 and 2 (*p* < 0.001). Collectively, these results indicate that the three terminal cell states identified by CellRank correspond to distinct positions along a continuous hepatocyte differentiation spectrum, spanning stemness‐associated states, intermediate maturation stages and functionally mature hepatocyte‐like states.

### 3.3 Transcriptional Regulatory Networks of HCC Cells Are Organized Into Functionally Distinct Regulatory Modules

Using single‐cell transcriptomic data, transcription factor–driven regulatory networks were reconstructed for hepatocytes and HCC cells based on the SCENIC framework. In addition to the core regulons defined by SCENIC, extended regulons were included to comprehensively capture transcriptional regulatory complexity. In total, 535 regulons were identified across the analysed cell populations.

**FIGURE 4:**
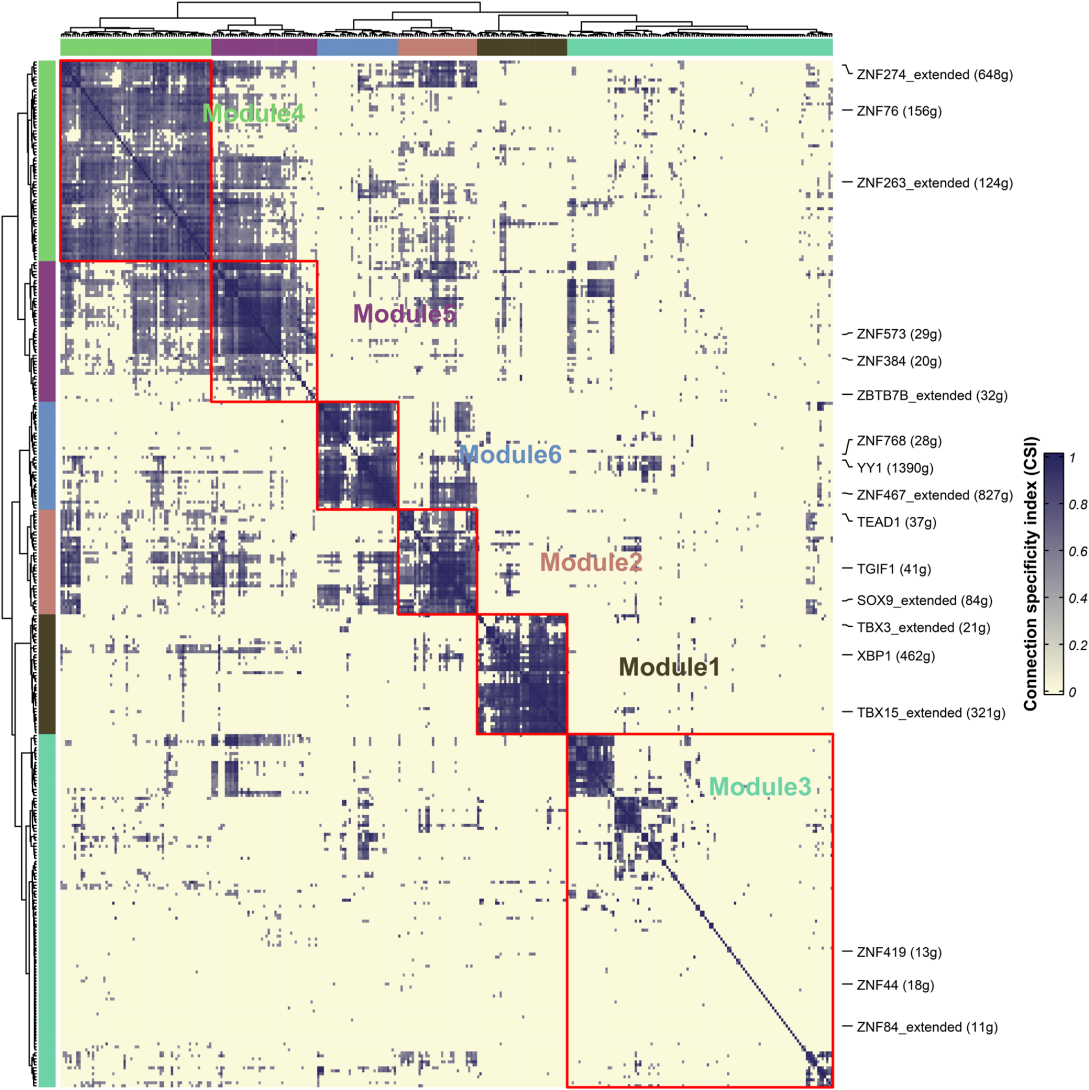
Heatmap of the connection specificity index (CSI) among regulons in HCC cells. This heatmap shows the hierarchical clustering of regulons based on the connection specificity index (CSI). Colour gradients from light to dark indicate increasing CSI values, with higher values reflecting stronger coordinated activity between regulons. Block‐like high‐CSI patterns are observed along the diagonal of the heatmap, corresponding to distinct regulatory modules (Module 1–Module 7). Within each module, regulons exhibit highly concordant activity patterns, whereas CSI values between modules are markedly lower, indicating functional separation among regulatory programs.

To characterize the organizational structure of the transcriptional regulatory network, the connection specificity index (CSI) was calculated for all regulon pairs, and a regulon similarity matrix was generated. Hierarchical clustering of the CSI matrix revealed a distinct modular architecture, as visualized by a CSI heatmap (Figure 4). The heatmap displayed pronounced block‐like patterns along the diagonal, indicating the presence of multiple regulatory modules with high internal similarity.

Within each module, regulon activity patterns showed strong concordance across the cell population, reflecting coordinated regulation by groups of transcription factors. In contrast, CSI values between different modules were substantially lower, suggesting functional separation among regulatory programs (Table S1). Moreover, representative regulons with high RSS values in each cell group were visualized to illustrate state‐specific activation patterns, highlighting key transcriptional programs selectively activated in specific cell populations (Figure S2). These modules, therefore, represent relatively independent and stable transcriptional regulatory units, each potentially associated with distinct cellular functional states.

Collectively, these results demonstrate that transcriptional regulation in HCC cells is highly organized and modular rather than governed by a single dominant regulator or linear regulatory axis. Cellular state heterogeneity arises from the coordinated activity of multiple functionally distinct regulatory modules, providing a structural basis for subsequent analyses linking terminal cell states to specific transcriptional regulatory programs.

### 3.4 State‐Specific Activity Patterns of Regulatory Modules Across Terminal Cell States

**FIGURE 5:**
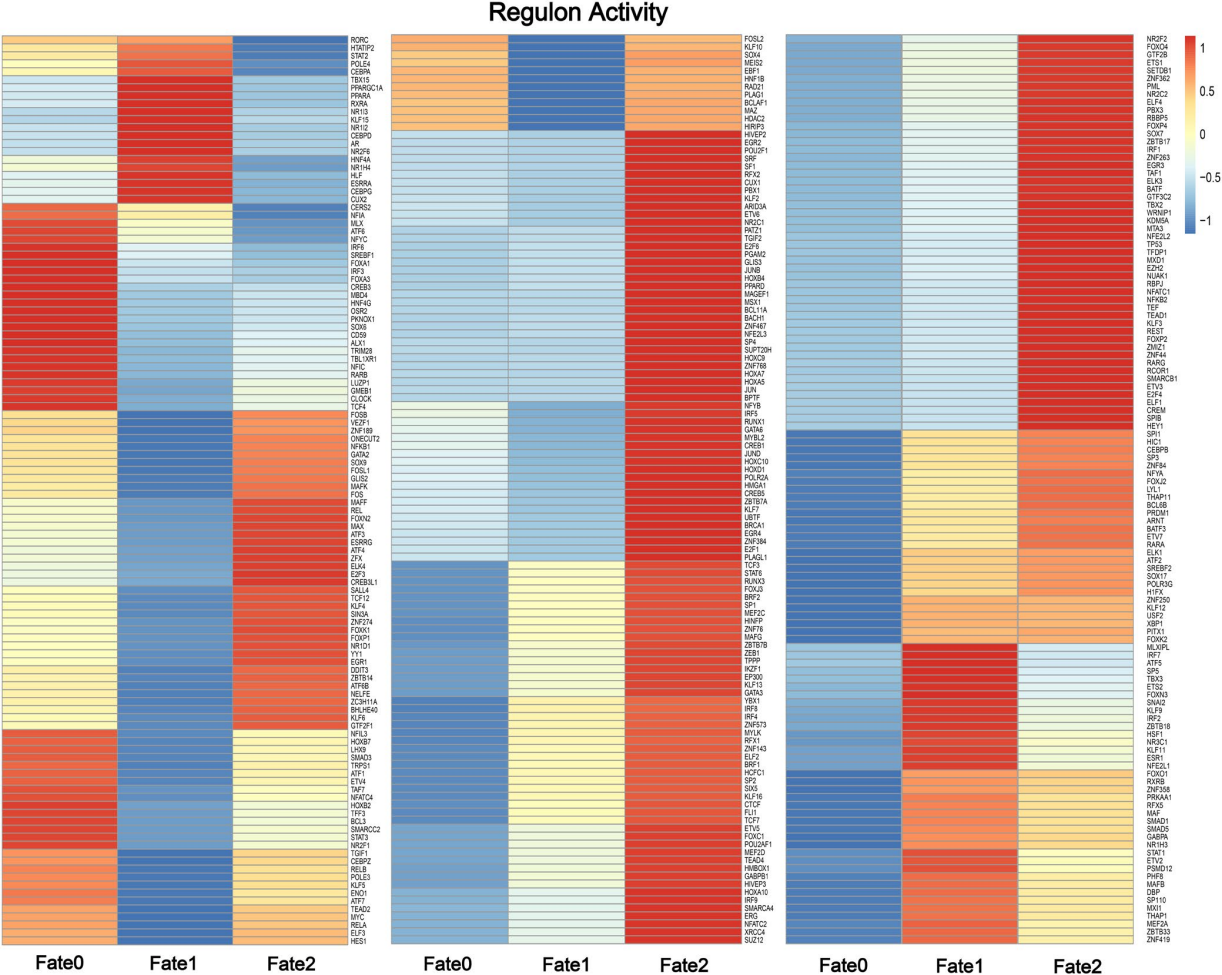
Regulon activity patterns across three terminal cell states. This heatmap displays the distribution of regulon activities across the three terminal cell fates (Fate 0, Fate 1, Fate 2) identified by CellRank. The activity levels are *Z*‐score normalized across fates, allowing comparison of relative activity between states. Colours range from blue (low activity) to red (high activity), reflecting the relative activation of each regulon in different terminal states. Rows represent regulons, while columns represent Fate 0 (stem‐like), Fate 1 (metabolic) and Fate 2 (proliferative) cells.

To characterize fate‐specific regulatory patterns, the average AUC values of each regulon were calculated within each of the three terminal cell fates and subjected to row‐wise normalization to capture relative activity differences across fates. The resulting heatmap revealed clearly separated regulon activity patterns among the terminal fates (Figure 5).

Most regulons within the same regulatory module exhibited selectively elevated activity in one terminal fate while remaining relatively low in the others, indicating a strong association between transcription factor–driven regulatory programs and specific cell fates (Figure 5). This fate‐preferential activation pattern suggests that distinct regulatory modules are selectively engaged during the stabilization of different terminal cell fates. In addition to these discrete patterns, a subset of regulons displayed gradual changes in activity across terminal fates, reflecting continuous regulatory transitions rather than strictly discrete boundaries. This graded behaviour indicates partial overlap in regulatory programs among fates and is consistent with a continuum of cell fate evolution in HCC.

Together, these results demonstrate that the three terminal cell fates identified by CellRank are not only distinguishable at the level of fate inference but are also characterized by distinct combinations of transcriptional regulatory programs. The concordance between cell fates and regulatory activity patterns supports the robustness of CellRank‐based fate classification and provides a regulatory context for linking transcription factors and regulatory modules to the functional characteristics of HCC cell states.

### 3.5 Distinct Terminal Cell States Are Governed by State‐Specific Transcriptional Regulatory Modules

To clarify the relationship between transcriptional regulatory networks and cell fate decisions, SCENIC‐reconstructed regulatory networks were integrated with CellRank derived fate‐driving information. In earlier analyses, fate‐specific high‐activity regulons were identified based on regulon activity profiles, providing a preliminary framework for potential fate‐associated regulatory networks. However, regulon activity alone is insufficient to distinguish regulators that play decisive roles in fate transitions. Therefore, CellRank‐inferred fate‐specific fate‐driving gene sets were further incorporated, and their intersection with high‐activity regulon target genes was used to identify core gene sets that are both transcriptionally active and directly associated with fate transitions. This integrative strategy yielded compact, fate‐specific regulatory networks for each terminal fate, substantially narrowing the pool of candidate regulators and providing a high‐confidence framework for dissecting core regulatory axes.

**FIGURE 6:**
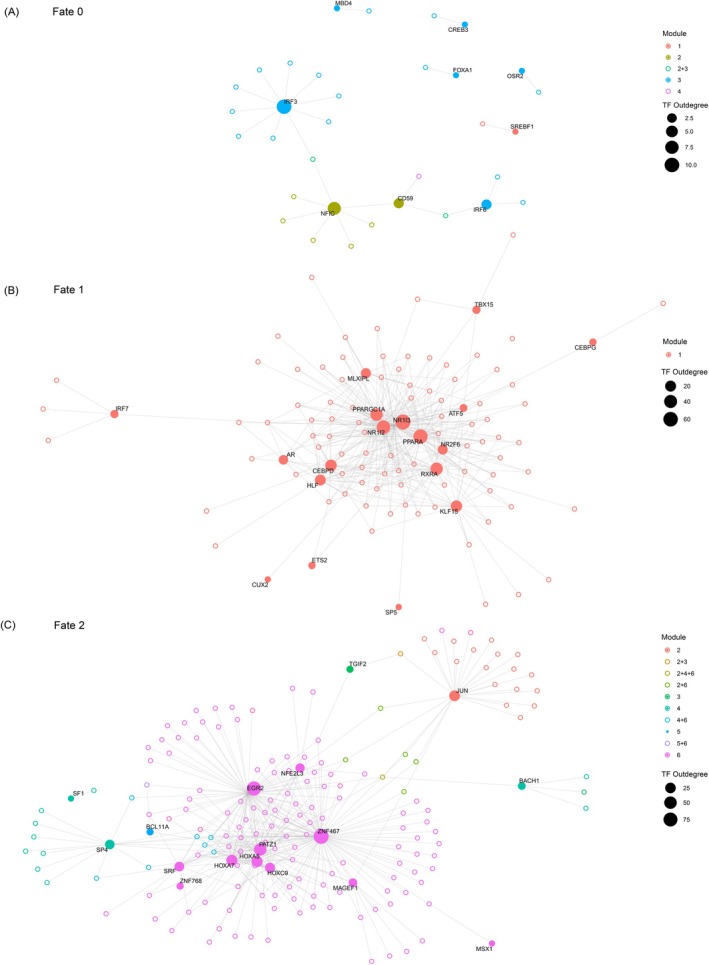
Core nodes and module associations in refined fate‐specific regulatory networks across three terminal cell fates. (A–C) Refined fate‐specific transcriptional regulatory networks for Fate 0, Fate 1, and Fate 2, respectively, highlighting core regulatory nodes and their module associations. Circular nodes represent transcription factors (TFs), whereas triangular nodes represent target genes. Node colours indicate regulatory modules (different colours correspond to different modules), and node size is positively correlated with TF outdegree, with larger nodes indicating higher regulatory connectivity. Edges represent regulatory relationships between TFs and their target genes. The Fate 0 network is predominantly associated with Module 3, the Fate 1 network shows enrichment in Modules 2 and 3, and the Fate 2 network is mainly concentrated in Modules 2, 4, and 6, illustrating distinct module preferences across terminal cell fates.

Refined fate‐specific transcriptional regulatory networks for the three terminal fates are shown in (Figure 6A–C, Tables S2 and S3). These networks highlight core regulatory nodes and their associated regulatory modules in Fate 0, 1 and 2, respectively. In each network, core nodes occupy central hub positions and exhibit high regulatory connectivity, suggesting critical roles in directing HCC cells toward specific terminal fates. Notably, the overall network architecture differs markedly across terminal fates, indicating that distinct fate outcomes are supported by different regulatory configurations rather than by a shared transcriptional backbone.

Integration of network visualization with regulon module classification further revealed pronounced module‐level preferences across terminal fates (Figure 6A–C). Key transcription factors within a given fate tended to cluster within one or a limited number of regulatory modules, forming highly interconnected core regulatory structures. This observation indicates that fate‐specific biological phenotypes are primarily achieved through selective activation or reinforcement of particular regulatory modules, rather than through global changes in transcription factor activity.

Consistent with the network‐level observations, a summary of dominant regulatory modules and representative core transcription factors for each terminal fate is provided in (Table 1). Fate 0 is primarily associated with Module 3, fate 1 with Module 1 and fate 2 with Module 6, each accompanied by a distinct set of key transcription factors. These differences reflect divergent transcriptional regulatory strategies across terminal fates and underscore the modular nature of fate control in HCC cells.

**TABLE 1:** Summary of dominant regulatory modules and key transcription factors across three terminal cell fates in hepatocellular carcinoma.

Fate	Main module	Key TFs
Fate 0 (immune‐activated)	Module 3	IRF3, IRF6
Fate 1 (metabolic)	Module 1	PPARA, NR1I3, CEBPD
Fate 2 (proliferative)	Module 6	EGR2, ZNF467,HOXA5

Collectively, these findings demonstrate that terminal cell fates are not governed by single master regulators but instead arise from the coordinated action of fate‐specific transcriptional regulatory modules. The combined network‐based visualization (Figure 6A–C) and tabular summary (Table 1) provide a coherent framework for understanding how distinct regulatory architectures support stable fate maintenance in hepatocellular carcinoma.

### 3.6 Regulatory Modules of Terminal Cell States Exhibit Distinct Functional Specialization

**FIGURE 7:**
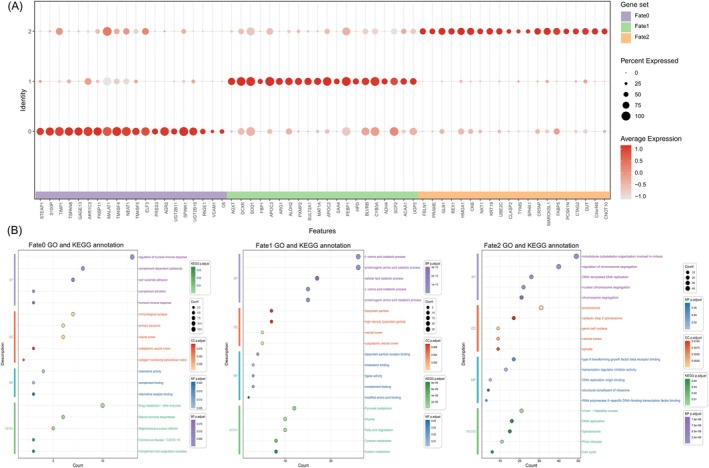
Expression patterns and functional enrichment of core regulatory nodes and fate‐driving genes across three terminal cell fates. (A) Dot heatmap showing expression patterns of core regulatory nodes and CellRank‐derived fate‐driving genes across the three terminal cell fates. Each row represents a gene, and columns correspond to terminal fates (fate 0, fate 1 and fate 2). Colour intensity indicates average gene expression levels (blue to red, low to high), while dot size reflects the proportion of cells expressing each gene within the corresponding fate. The coloured annotation bar indicates fate assignment of genes. Distinct fate‐specific expression patterns are observed at both the expression level and prevalence level. (B) Bubble plots of Gene Ontology (GO) enrichment analysis for the three terminal cell fates (from left to right: Fate 0, fate 1 and fate 2). The x‐axis indicates the number of enriched genes, and the y‐axis represents enriched GO terms. Bubble size reflects gene ratio, and colour denotes functional categories. Fate 0 is predominantly enriched for immune‐ and inflammation‐related processes, fate 1 for metabolism‐ and detoxification‐related functions, and fate 2 for cell cycle– and transcription‐related processes.

GO functional annotation and KEGG pathway enrichment analyses were performed on core transcription factors and their associated CellRank‐derived fate‐driving genes for each terminal cell fate. The enrichment results revealed clearly distinct functional profiles across the three cell fates (Figure 7).

In addition to functional enrichment analysis, the expression patterns of core regulatory nodes and CellRank‐derived fate‐driving genes were examined across the three terminal cell fates (Figure 7A). Distinct fate‐specific expression profiles were observed at both the expression level and the proportion of expressing cells. Core driver genes associated with Fate 0, Fate 1 and Fate 2 showed preferential expression within their corresponding terminal fates, with limited overlap across fates. This clear stratification of gene expression supports the robustness of CellRank‐based fate classification and indicates that terminal cell fates are characterized by coherent and fate‐specific transcriptional programs.

Cells in Fate 0 (immune‐activated hepatocytes) showed functional enrichment predominantly associated with immune and inflammatory response–related pathways (Figure 7B, **left**). Significantly enriched pathways included complement and coagulation cascades, cytokine–cytokine receptor interactions, and humoral immune regulation, along with pathways related to viral and bacterial infections. This enrichment pattern indicates that Fate 0 cells are characterized by activation of immune‐responsive transcriptional programs, reflecting a functional state oriented toward inflammatory responses and host defence rather than metabolic homeostasis.

Cells in Fate 1 (metabolic hepatocytes) exhibited enrichment patterns characteristic of core hepatocyte metabolic functions (Figure 7B, **middle**). Central metabolic pathways—including amino acid metabolism, lipid metabolism, carbohydrate metabolism, and energy metabolism—were significantly enriched, encompassing glycolysis, the tricarboxylic acid cycle, fatty acid degradation, cholesterol and steroid metabolism, as well as multiple amino acid synthesis and degradation pathways. In addition, liver‐specific detoxification pathways and transport‐related signalling processes showed enhanced enrichment. Together, these features highlight the dominant role of Fate 1 cells in maintaining metabolic homeostasis and liver‐specific metabolic and detoxification functions.

In contrast, cells in Fate 2 (proliferative hepatocytes) were characterized by functional enrichment dominated by proliferative and transcriptional activity–related pathways (Figure 7B, **right**). Significantly enriched pathways included cell cycle regulation, DNA replication, ribosome biogenesis, and spliceosome function, accompanied by activation of pathways associated with transcriptional regulation and protein synthesis. These enrichment patterns indicate that Fate 2 cells possess elevated proliferative activity and extensive transcriptional remodelling, consistent with a highly dynamic cellular state distinct from functionally mature hepatocytes.

### 3.7 Clinical Relevance of the Proliferative (Fate 2) Regulatory Signature in HCC

Given that the Fate2 hepatocyte state is characterized by enhanced proliferative activity and enrichment of malignant features at the single‐cell level, representing a biologically aggressive subpopulation of hepatocellular carcinoma cells, this section focuses on the Fate2 state and its associated transcriptional regulatory network to evaluate its clinical relevance at the patient level.

**FIGURE 8:**
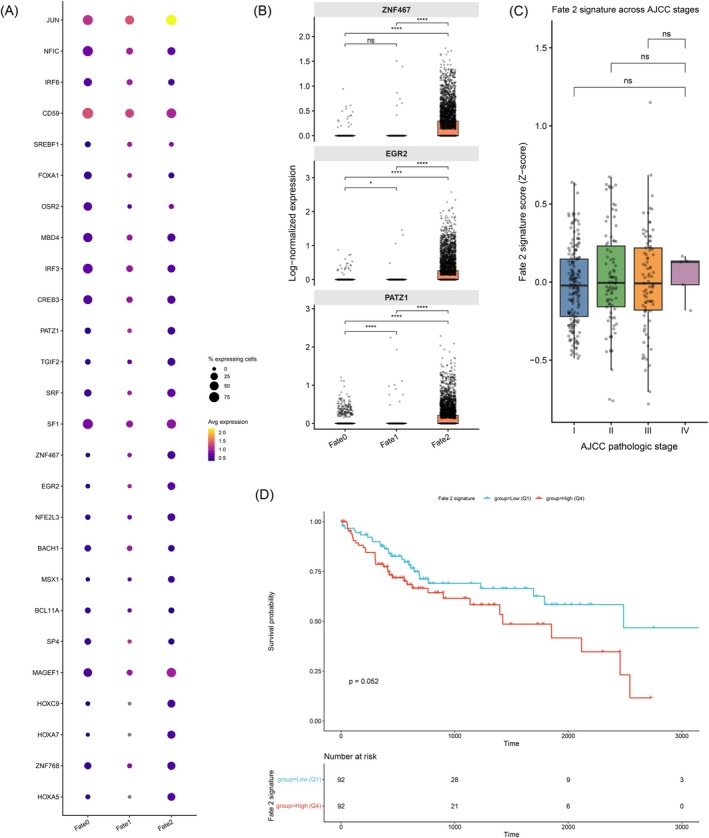
Activation of the Fate2 regulatory network and its clinical relevance. (A) Expression prevalence and expression intensity of core transcription factors from the Fate2 regulatory network across Fate0 (immune‐activated), Fate1 (metabolic), and Fate2 (proliferative) hepatocyte states at the single‐cell level. Transcription factors generally exhibit higher proportions of expressing cells in Fate0 and Fate2 compared with Fate1, with JUN showing particularly elevated expression activity in Fate2 cells. (B) Gene‐level expression comparison of representative Fate2 regulatory transcription factors (ZNF467, EGR2, and PATZ1) across different Fate states, demonstrating higher expression in Fate2 cells relative to Fate0 and Fate1 cells. (C) Distribution of the Fate2 signature score across AJCC pathological stages in the TCGA‐LIHC cohort. No statistically significant differences were observed among stages. (D) Kaplan–Meier analysis of overall survival in TCGA‐LIHC patients stratified by Fate2 signature score, comparing high (Q4) and low (Q1) expression groups. Patients with higher Fate2 signature scores tended to exhibit poorer overall survival.

At the single‐cell level, expression patterns of core transcription factors within the Fate2 regulatory network were compared across Fate0, Fate1 and Fate2 hepatocyte states (Figure 8A). These transcription factors showed markedly higher proportions of expressing cells in Fate0 and Fate2 than in Fate1 cells, highlighting a clear difference in transcriptional regulatory activity between the metabolic Fate1 state and the immune‐activated Fate0 and proliferative Fate2 states. This pattern indicates that Fate1 hepatocytes maintain a relatively stable metabolic phenotype, whereas Fate0 and Fate2 cells display more active and dynamic transcriptional programs.

Within the Fate2 regulatory network, JUN showed particularly prominent behaviour. Both the proportion of JUN‐expressing cells and its expression level were higher in Fate2 cells compared with Fate0 and Fate1 cells (Figure 8A), consistent with enhanced activation of AP‐1–related transcriptional programs in proliferative malignant hepatocytes.

To further substantiate activation of the Fate2 regulatory program, representative transcription factors (ZNF467, EGR2, and PATZ1) were examined at the gene level. All three genes exhibited significantly higher expression in Fate2 cells than in Fate0 and Fate1 cells (Figure 8B), supporting global activation of the Fate2 transcriptional network.

At the patient level, a Fate2 signature was constructed using 26 transcription factors from the Fate2 regulatory network and evaluated in the TCGA‐LIHC cohort. No significant differences in Fate2 signature scores were observed across AJCC pathological stages (Figure 8C). This may be partly due to the limited number of stage IV patients in the cohort. In addition, hepatocellular carcinoma exhibits high intratumoral heterogeneity, with diverse subpopulations of malignant hepatocytes coexisting within tumours of the same stage. As a result, the proportion of Fate2‐like cells may vary independently of overall tumour stage, potentially masking stage‐dependent trends in signature scores. These observations highlight that cell‐state–specific transcriptional programs can provide complementary prognostic information beyond traditional pathological staging.

Survival analyses indicated a consistent trend toward poorer outcomes associated with higher Fate2 signature scores. n the univariable Cox model, each 1‐standard‐deviation increase in the Fate2 signature score was associated with an approximately 88% higher hazard of death (HR = 1.88, 95% CI 0.99–3.56, *p* = 0.050; Table 2). This association was attenuated but remained directionally consistent after adjustment for AJCC stage and age (HR = 1.75, 95% CI 0.90–3.38, *p* = 0.097; Table 2). Kaplan–Meier analysis further showed that patients with high Fate2 signature scores (Q4) tended to have worse overall survival compared with those with low scores (Q1) (Figure 8D; log‐rank *p* = 0.052).

**TABLE 2:** Cox regression analysis of Group 2 signature for OS in TCGA‐LIHC.

Endpoint	Model	Variable	HR	95% CI	*p*
OS	Univariate	Fate 2 signature (*Z*‐score)	1.88	1.00–3.56	0.050
OS	Multivariate	Fate 2 signature (*Z*‐score)	1.75	0.90–3.38	0.097
OS	Multivariate	Stage II vs. I	1.36	0.86–2.23	0.218
OS	Multivariate	Stage III vs. I	2.73	1.79–4.17	< 0.001
OS	Multivariate	Stage IV vs. I	5.08	1.56–16.49	0.0069
OS	Multivariate	Age	1.01	1.00–1.03	0.102

## 4 Discussion

HCC exhibits pronounced cellular heterogeneity and a highly complex evolutionary process, with distinct tumour cell states differing substantially in transcriptional features, functional properties, and potential fate trajectories. Although bulk transcriptome–based studies have identified several key driver genes and signalling pathways, they are inherently limited in resolving dynamic transitions among heterogeneous cell states and the underlying regulatory mechanisms. By integrating single‐cell transcriptomic data with cell fate inference and transcriptional regulatory network reconstruction, the present study systematically characterizes HCC cell state heterogeneity from both dynamic and mechanistic perspectives, providing new insights into HCC initiation and progression.

Accurate identification of malignant cells is essential for downstream single‐cell analyses. In this study, InferCNV‐based copy number variation inference enabled discrimination between malignant and non‐malignant cell populations at single‐cell resolution, yielding a relatively reliable set of tumour cells for subsequent analyses. Harmony‐based batch correction further reduced technical noise across samples, allowing tumour cells from different tissue origins to be analysed within a unified low‐dimensional space. This preprocessing strategy supported robust cell fate inference and regulatory network reconstruction and improved the stability and comparability of downstream results.

At the level of cell fate dynamics, CellRank was applied to model transcriptional state transitions of tumour cells and identified three terminal cell fates with distinct biological characteristics. Unlike conventional pseudotime analyses that primarily describe linear trajectories, CellRank employs a Markov chain–based framework to model transition probabilities in state space and can capture multi‐branch fate decisions. These results indicate that HCC cells do not evolve along a single trajectory but instead diverge toward multiple terminal fates associated with distinct functional features. To further characterize differentiation differences among terminal fates, five hepatocyte differentiation–related gene programs (HB1, HB2, FH1, FH2 and AH) were incorporated and quantified at single‐cell resolution using AUCell [8]. These programs exhibited marked differences in activity across the three terminal fates and collectively reflected a continuum from high‐stemness states toward mature hepatocyte‐like phenotypes, providing functional support for the fate structure inferred by CellRank.

At the transcriptional regulatory level, SCENIC was used to reconstruct transcription factor–gene regulatory networks and quantify regulon activity across cell states. Compared with analyses based solely on differentially expressed genes, SCENIC identifies putative transcriptional regulatory units at single‐cell resolution and provides a closer approximation of the underlying regulatory architecture. Fate‐specific analysis of regulon activity revealed relatively independent regulatory programs associated with distinct terminal fates, suggesting that tumour cells rely on different transcriptional strategies during fate differentiation. By integrating CellRank‐derived fate‐driving genes with fate‐specific regulon networks, this study further identified key regulatory factors that are both transcriptionally active and directly associated with fate transitions. Construction of refined fate‐specific regulatory networks and calculation of multiple topological metrics (including in‐degree, out‐degree, betweenness centrality and clustering coefficient) enabled identification of core regulatory nodes occupying central network positions. These core regulators may contribute to driving or maintaining specific cell fates and represent candidate targets for further mechanistic investigation.

Consistent with the functional characteristics of the three terminal fates, core regulatory factors showed distinct fate‐associated roles. The regulatory network of Fate 0 is centred on IRF3, NFIC and CD59 and is functionally focused on immune and inflammatory responses. IRF3 is a core component of the cGAS–STING signalling pathway, a central axis of innate immune responses. Previous studies have shown that IRF3 knockdown can reverse ionizing radiation–induced, T lymphocyte–mediated antitumor effects, and that this signalling pathway can influence hepatic inflammation through autophagy‐ and metabolism‐related mechanisms, supporting the interpretation that Fate 0 cells reside in an immune‐activated state [14, 15]. NFIC has been implicated in DNA hydroxymethylation processes and, together with GATA family transcription factors, contributes to the development of alpha‐fetoprotein (AFP)‐negative HCC [16]. CD59, a complement regulatory protein, exhibits restricted expression in HCC patients; its enrichment in Fate 0 suggests that these cells may participate in immune microenvironment remodelling through modulation of the complement pathway [17].

The regulatory network of Fate 1 is organized around NR1I3, PPARA, TBX15, KLF15, NR1I2, and CEBPD, forming a dense regulatory module consistent with a “mature hepatocyte homeostasis–maintaining” phenotype. NR1I3 has been experimentally validated as a target of lncRNA F11‐AS1, which upregulates NR1I3 expression by sponging miR‐211‐5p, thereby suppressing proliferation, migration, and invasion of HBV‐related HCC cells. Low expression of F11‐AS1 is associated with poor patient prognosis, and enrichment of NR1I3 suggests that Fate 1 cells retain aspects of normal hepatocyte function [18]. PPARA expression at both RNA and protein levels is significantly reduced in HCC tissues compared with normal liver and is closely associated with clinicopathological features and prognosis. Mechanistically, PPARA inter‐acts with ferroptosis‐related core factors such as GCLC to coordinately regulate ferroptosis, thereby maintaining lipid metabolism and detoxification functions [19]. TBX15 functions as a tumour suppressor; promoter hypermethylation leads to reduced mRNA expression, and low TBX15 expression is associated with significantly de‐creased 5‐year overall survival, serving as an independent prognostic factor. TBX15 is proposed to maintain differentiation homeostasis by suppressing reactive oxygen species (ROS) signalling while also modulating the NF‐κB pathway to contribute to an immunosuppressive microenvironment [20]. KLF15 is downregulated in HCC and inhibits tumour proliferation by transcriptionally activating PDLIM2, thereby suppressing the NF‐κB pathway and reducing lipid droplet formation [21]. NR1I2 is a key regulator of PCSK9 expression and may promote immune suppression in HCC through transcriptional activation of PCSK9 [22]. CEBPD expression is negatively correlated with ROCK2; ROCK2 suppresses CEBPD expression via phosphorylation of the GSK3β/β‐catenin pathway, thereby relieving inhibition of HCC cell proliferation [23]. Collectively, these transcription factors support the core phenotype of Fate 1 as metabolically stable and functionally mature hepatocytes.

The regulatory network of Fate 2 is organized around EGR2, ZNF467, PATZ1, HOXA7, HOXC9, and HOXA5, with functions focused on stemness maintenance and malignant proliferation. EGR2 is a Fate 2–specific high‐activity transcription factor whose expression level is positively correlated with cellular proliferative capacity; its downregulation significantly suppresses colony formation and tumour growth, directly reflecting the strong stemness features of this state [24]. ZNF467 regulates adipogenesis‐related genes to maintain stem cell lineage commitment, suggesting that Fate 2 cells reside in a dedifferentiated functional stage [25]. High expression of RRP15 upregulates PATZ1, which subsequently activates the LAMC2/FAK signalling pathway, ultimately promoting epithelial–mesenchymal transition (EMT) and migratory capacity [26]. HOXA7 directly regulates cyclin E1/CDK2 protein expression, driving cell cycle progression and proliferation [27]. HOXC9 is highly expressed in HCC and associated with poor prognosis; its knockdown significantly inhibits cell proliferation and invasion and also modulates the tumour microenvironment through immune gene regulation [28]. HOXA5 is downregulated in HCC tissues and is associated with increased tumour size and poor prognosis; its knockdown promotes angiogenesis and tumour growth. As a direct target of miR‐130b‐3p, HOXA5 participates in the Sp1/miR‐130b‐3p/HOXA5 regulatory axis to drive HCC progression [29]. Together, the coordinated activity of these transcription factors supports the Fate 2 phenotype characterized by enhanced proliferative capacity and pronounced stemness features. More broadly, these transcription factors do not act in isolation but operate within a coordinated HOX‐centered regulatory program that sustains stemness‐associated malignant states in HCC. Integrative bioinformatics analyses of TCGA and ICGC cohorts have demonstrated that high activity of HOX modules is associated with enhanced cell‐cycle progression, self‐renewal capacity and therapy resistance, as well as remodelling of the tumour microenvironment and altered drug sensitivity [30, 31]. These findings suggest that the cooperative activity of HOX family transcription factors underlies the aggressive phenotype and poor prognosis observed in hepatocellular carcinoma.

In summary, the core transcription factors identified for each terminal state act collectively to establish distinct functional phenotypes. Fate 1 is characterized by transcriptional regulators that maintain hepatocyte homeostasis and metabolic stability, reflecting a more mature, less malignant state. In contrast, Fate 2 is dominated by HOX‐family and proliferation‐associated transcription factors that reinforce stemness, cell‐cycle progression, and malignant proliferation. Fate 0 occupies an intermediate position with mixed regulatory programs. These findings suggest that each terminal state is defined not by single genes, but by coordinated regulatory networks that shape specific cellular behaviours and contribute to the differential malignant potential observed among HCC subpopulations.

Despite the comprehensive integration of single‐cell fate inference and transcriptional regulatory network analysis, several limitations should be acknowledged. First, regulatory network reconstruction was primarily based on transcriptomic data and did not fully account for epigenetic modifications, post‐translational regulation, or spatial microenvironmental influences on cell fate. Second, inferCNV and CellRank inferences are partially dependent on parameter settings and reference cell selection, and future validation using independent cohorts and experimental approaches is warranted. Finally, the key regulatory factors identified in this study require further in vitro and in vivo validation to elucidate their precise functional roles in HCC development and progression. Additionally, spatial microenvironmental influences on cell fate were not fully captured in this study, and future work integrating spatial transcriptomics may further refine understanding of HCC cellular heterogeneity.

## 5 Conclusions

In conclusion, by integrating single‐cell fate inference with transcriptional regulatory network analysis, this study establishes a unified framework to dissect terminal cell fates in hepatocellular carcinoma. Three distinct terminal fates—immune‐activated, metabolic and proliferative hepatocytes—were identified along a continuous differentiation spectrum, each characterized by specific regulatory modules and core transcription factors.

These findings provide mechanistic insight into HCC cellular heterogeneity from a dynamic and regulatory perspective and highlight fate‐specific regulatory programs as potential targets for precision therapeutic strategies.

## Author Contributions

**Yun Zhu:** software, data curation. **Biaolong Deng:** conceptualization, methodology, formal analysis, writing – original draft. **Wenhao Lin:** software, visualization. **Ke Xu:** methodology, software, writing – original draft, writing – review and editing, visualization. **Tao Yang:** methodology, supervision. **Yuanyuan Chen:** conceptualization, supervision, funding acquisition, writing – review and editing. **Xiaoyan Zhou:** conceptualization, supervision, writing – review and editing. **Lang Lu:** methodology.

## Funding

This research was funded by the Fundamental Research Funds for the Central Universities (YDZX2025015).

## Conflicts of Interest

The authors declare no conflicts of interest. The funders had no role in the design of the study; in the collection, analysis or interpretation of data; in the writing of the manuscript; or in the decision to publish the results.

## Supporting information


**Table S1:** Cluster assignment of regulons in hepatocytes and HCC cells.
**Table S2:** Edge list of fate‐specific transcriptional regulatory networks in HCC cells.
**Table S3:** Node information for the fate‐specific transcriptional regulatory networks, including transcription factor identity, topological metrics, module assignment and core status.

## Data Availability

Publicly available datasets were analyzed in this study. Single‐cell RNA sequencing data were obtained from the Gene Expression Omnibus (GEO) database under accession number GSE149614. Bulk RNA sequencing data, together with corresponding clinical and survival information, were obtained from The Cancer Genome Atlas Liver Hepatocellular Carcinoma (TCGA‐LIHC) cohort via the UCSC Xena platform.
